# Characteristics of sleep/wake problems and delivery outcomes among pregnant Japanese women without gestational complications

**DOI:** 10.1186/s12884-020-02868-1

**Published:** 2020-03-20

**Authors:** Shiho Umeno, Chiho Kato, Yuki Nagaura, Hideaki Kondo, Hiromi Eto

**Affiliations:** 1 Department of Reproductive Health, Nagasaki University, Institute of Biomedical Sciences, 1-7-1 Sakamoto, Nagasaki, 852-8520 Japan; 2 Karatsu Red Cross Hospital, 2430 Watada, Karatsu, Saga 847-8588 Japan; 3 Department of Maternal Nursing/Midwifery, Japanese Red Cross College of Nursing, 4-1-3 Hiroo, Shibuya-ku, Tokyo, 150-0012 Japan; 4 Department of General Medicine, Nagasaki University Graduate School of Biomedical Sciences, 1-7-1 Sakamoto, Nagasaki, 852-820 Japan; 5 International Institute for Integrative Sleep Medicine, University of Tsukuba, 1-2 Kasuga, Tsukuba, Ibaraki 305-8550 Japan

**Keywords:** Excessive daytime sleepiness, Insomnia, Sleep disordered breathing, Sleep quality, Restless legs syndrome/Willis-Ekbom disease

## Abstract

**Background:**

Frequently observed sleep/wake problems among pregnant women need comprehensive evaluation. This study was conducted to clarify the sleep/wake problems among pregnant women without gestational complications during the second and third trimester and the effects of sleep/wake problems on delivery outcomes.

**Methods:**

A total of 88 Japanese pregnant women participated in this study. In their second and third trimester, subjective sleep quality, insomnia severity, excessive daytime sleepiness (EDS), and restless legs syndrome/Willis-Ekbom disease (RLS/WED) were assessed using questionnaires; also, sleep disordered breathing (SDB) was screened using a pulse oximeter.

**Results:**

From the second to the third trimester, an increasing tendency of sleep/wake problems was observed. During the third trimester, the percentages of women experiencing decreased subjective sleep quality, difficulty maintaining sleep (DMS), EDS, RLS/WED, and 3% oxygen desaturation index (ODI) values ≥5/h were 62.5, 45.5, 48.9, 9.1, and 29.5%, respectively. In a logistic regression analysis for EDS in the third trimester, the adjusted odds ratio (95% confidence interval) of total sleep duration < 6 h, moderate to severe DMS, and 3% ODI values ≥5/h were 3.25 (1.16–9.10), 4.74 (1.60–14.00), and 0.90 (0.28–2.89), respectively. Although short sleep durations, decreased subjective sleep quality, EDS, and SDB did not affect delivery outcomes or the infant’s condition, the percentage of women undergoing cesarean sections in the severe insomnia group was significantly higher (*p* = 0.008).

**Conclusions:**

Sleep/wake problems were frequent during pregnancy, especially during the third trimester. EDS among pregnant women was associated with shorter sleep durations and DMS rather than SDB. The effect of factors related to insomnia on delivery outcomes should thus be considered a crucial problem among pregnant Japanese women without gestational complications in clinical practice.

## Background

Sleep/wake problems are frequently observed among pregnant women. During pregnancy, increased waking after sleep onset and decreased sleep efficiency are common findings [1, 2], and complaints about insomnia symptoms are prevalent [3]. Additionally, a decrease in subjective sleep quality is often observed [4] and complaints about excessive daytime sleepiness (EDS) are frequent among pregnant women [5]. Moreover, sleep disordered breathing (SDB) and restless legs syndrome/Willis-Ekbom disease (RLS/WED) are also prevalent. The prevalence of SDB and that of RLS/WED both increase during the course of pregnancy, with meta-analyses reporting a prevalence of 15% (95% confidence interval [CI]: 12–18%) [6] and of 21% (95% CI: 17–25) [7], respectively.

Sleep/wake problems might be associated with gestational complications and delivery outcomes. SDB, RLS/WED, shorter or longer sleep durations, poor sleep quality, and EDS have been reported to be associated with gestational diabetes and hypertensive disorders during pregnancy [6, 8–12]. Moreover, these maternal sleep/wake problems affect cesarean delivery, preterm birth rates, rates of children born small- and large-for-gestational-age, and admission rates to the neonatal intensive care unit [6, 8, 11–14]. However, findings are inconsistent, and discrepancies among studies are thought to be influenced by methodological differences across studies [6, 8, 12, 13, 15]. In addition, mutual interactions among different types of sleep/wake problems cannot be ignored.

To clarify the impact of sleep/wake problems on delivery outcomes, sleep/wake problems must be evaluated comprehensively. This study thus aimed to assess the sleep/wake problems among pregnant Japanese women without gestational complications during the second and third trimester of pregnancy as well as the effects of these sleep/wake problems on delivery outcomes.

## Methods

### Participants and protocol

**Fig. 1:**
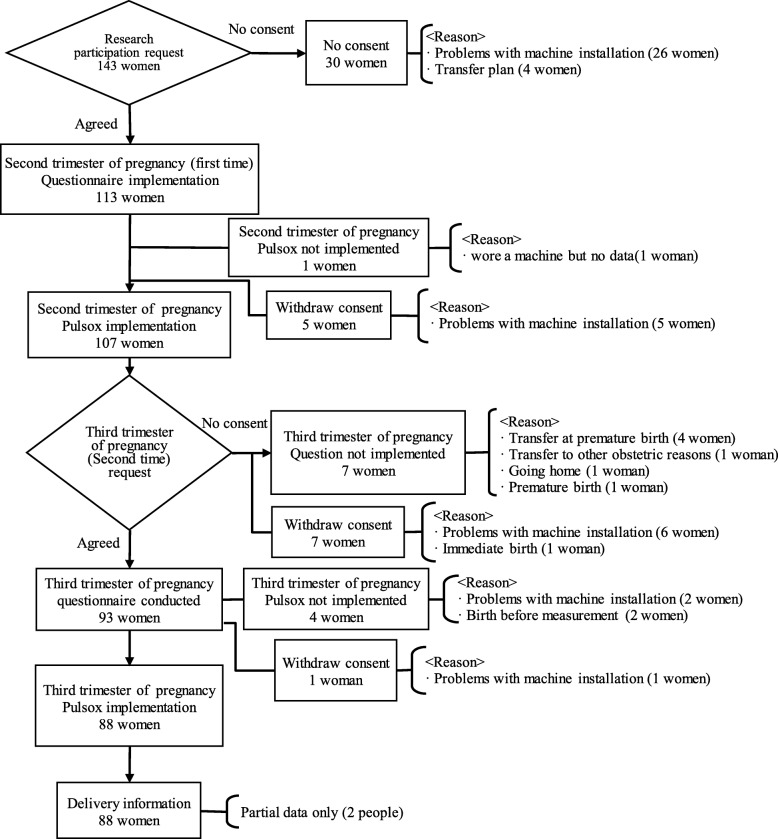
Data collection algorithm

The participants were pregnant women who were undergoing pregnancy-related medical examinations at an obstetric medical facility in Nagasaki Prefecture between December 2017 and October 2018. Women who had pregnancy complications were excluded. A total of 143 pregnant women were asked to participate in the study and 113 women consented to participate in their second trimester of pregnancy (around 24 weeks gestation). After accounting for women who dropped out due to obstetric complications or technological issues related to data collection during the study period, valid responses were obtained from 107 women (74.8%) in the second trimester and 88 (61.5%) in the third trimester (around 37 weeks gestation). The final analysis was performed in 88 participants (Fig. 1).

All procedures performed in this study involving human participants were in accordance with the ethical standards of the institutional and/or national research committee and with the 1964 Helsinki declaration and its later amendments or comparable ethical standards. This study was approved by the Ethics Committee of Nagasaki University Graduate School of Biomedical Sciences (approval no. 1711090). Written informed consent was obtained from all individual participants included in the study.

We obtained demographic and clinical characteristics of all participants including age, body weight (pre-pregnancy, second trimester, and third trimester), height, history of gravidity, and history of parity. Pre-pregnancy, second-trimester, and third-trimester BMIs were calculated. The delivery and neonatal information were collected from midwifery records and included gestational age, duration of birth, type of birth (vaginal delivery, cesarean section), labor induction, episiotomy, perineal laceration, uterus contraction, oxytocic agent use, volume of blood loss, the infant’s condition, infant weight, infant height, Apgar score, umbilical pH, and umbilical partial pressure of carbon dioxide. Self-reported questionnaires related to sleep/wake problems were administered and SDB screening was performed over 2 consecutive nights during the second and third trimester using a pulse oximeter.

### Pulse oximeter

SDB was screened using a PULSOX-300i (KONICA MINOLTA Japan, Inc., Tokyo, Japan). The pulse oximeter was attached to the first joint of the second or third fingers of the non-dominant hand at bedtime and removed at the time of awakening over 2 consecutive nights. The data were downloaded to a personal computer using DS-Me version 2.1 (KONICA MINOLTA Japan, Inc.). After removing poor measurement periods, the 3% ODI was calculated, defined as the number of times per hour in which the oxygen saturation decreased by 3% or more from the baseline. Patients who had a 3% ODI ≥ 5/h were defined as having suspected SDB [16].

### Questionnaires

#### Pittsburgh sleep quality index

Subjective sleep quality was assessed using the Japanese version of the Pittsburgh Sleep Quality Index (PSQI). This questionnaire consists of seven components, including sleep quality (C1), sleep latency (C2), sleep duration (C3), sleep efficiency (C4), sleep disturbance (C5), hypnotic use (C6) and daytime dysfunction (C7). The score of each component ranges from 0 to 3, with global scores ranging from 0 to 21. Higher scores indicate inadequate sleep quality with scores ≥6 indicating poor sleep quality [17, 18]; therefore, women with global scores ≥6 were categorized into a “poor sleeper” group and women with each component score ≥ 2 were categorized into a “worsened condition” groups. According to a meta-analysis, the sensitivity and specificity (95% CI) for insomnia are 0.94 (0.86–0.98) and 0.76 (0.64–0.85), respectively [19].

#### Insomnia severity index

Insomnia severity was assessed using the Japanese version of the Insomnia Severity Index (ISI). Questions regarding the participants’ level of difficulty initiating sleep (DIS) and difficulty maintaining sleep (DMS) are answered with “none”, “mild”, “moderate”, “severe”, or “very severe”. All responders who had “mild” to “very severe” symptoms were defined as having DIS and DMS. Responders who had “moderate” to “very severe” symptoms were defined as having moderate to severe DIS and DMS. The global score of this index ranges from 0 to 28, with higher scores indicating greater insomnia severity, and the cut off score for insomnia is 10 points [20]. According to a meta-analysis, the sensitivity and specificity (95% CI) for insomnia are 0.88 (0.79–0.93) and 0.85 (0.68–0.94), respectively [19].

#### Epworth sleepiness scale

Daytime sleepiness was assessed using the Japanese version of the Epworth Sleepiness Scale (ESS). The global score on this scale ranges from 0 to 24, with higher scores indicating greater subjective daytime sleepiness and the cut off score for EDS is 10 points [21, 22]. In this study, we categorized participants with an ESS score ≥ 11 as the EDS group, because a daytime dysfunction score ≥ 2 on the PSQI, which reflects daytime sleepiness, was associated with an ESS score ≥ 11, not an ESS score ≥ 10.

#### Cambridge-Hopkins questionnaire short form 13

Symptoms related to RLS/WED were assessed using the Japanese version of the Cambridge-Hopkins questionnaire short form 13 (CH-RLSq13). The CH-RLSq13 is a self-reported questionnaire containing 13 items, 10 of which are related to characteristic symptoms (i.e., an urge to move the legs, which is usually but not always accompanied by uncomfortable and unpleasant sensations during period of rest, that is worse in the evening or night and relieved by movement) and the exclusion of other conditions (e.g., leg cramping and positional discomfort); the remaining three items are related to symptom severity and onset. The sensitivity and specificity of the original CH-RLSq13 for an RLS/WED diagnosis have been reported as 87.2 and 94.4%, respectively, and those of the Japanese version are 88.9 and 100.0%, respectively [23].

### Statistical analysis

The prevalence of SDB among pregnant women has been reported to be 15% [6]. In the present study, we assumed the same prevalence of SDB and a 95% confidence interval of 15%. To confirm the prevalence of SDB in our participant population, an estimated sample size of 87 was required. Additionally, the prevalence of RLS/WED among pregnant women has been reported to be 21% [7]. In the present study, we assumed prevalence of RLS/WED of 20% and a 95% confidence interval of 20%. To confirm the prevalence of RLS/WED in our participant population, a sample size of 61 was thus required.

R version 3.5.2 and EZR version 1.40 (http://www.jichi.ac.jp/saitama-sct/SaitamaHP.files/statmed.html) [24] were used for statistical analyses. Categorical variables were presented as counts and percentages. Continuous variables were presented as the mean and SD when normally distributed and as medians and IQRs when non-normally distributed. Comparisons were made using *t*-tests for normally distributed data and Mann-Whitney *U* tests for non-normally distributed data. Comparisons of continuous variables between the second and third trimester were performed using the Wilcoxon signed-rank test. Frequency analyses for categorical data were performed using Fisher’s exact test. Frequency analyses between the second and third trimester were performed using McNemar’s test. The two-sided alpha level was set at 0.05.

The odds ratios for EDS were calculated using logistic regression analysis to assess associations with total sleep durations, DMS, and SDB. After a univariate analysis, adjustments for age and BMI were made. Age and BMI were categorized into two groups each: age < 30 years (reference) or ≥ 30 years and BMI < 25 kg/m^2^ (reference) or ≥ 25 kg/m^2^. Total sleep durations obtained from the PSQI were also categorized into two groups: ≤ 6 h and > 6 h (reference). DMS was categorized into none-mild (reference) and moderate-severe, and 3% ODI values were categorized into < 5/h (reference) and ≥ 5/h.

## Results

Baseline characteristics are shown in Table 1. The participants were between the ages of 19 and 42 years, with a mean age ± SD of 30.9 ± 4.7 years. The data were collected at 24.6 ± 0.6 weeks of gestation in the second trimester and 36.2 ± 0.9 weeks of gestation in the third trimester (Additional file 1).

**Table 1:** Baseline characteristics of study participants

N	88
Age years, mean ± SD	30.9 ± 4.7
Primipara, n (%)	40 (46.0)
BMI kg/m^2^, median (IQR)^a^	20.3 (18.9–22.1)
< 18.5 kg/m^2^, n (%)	16 (18.4)
18.5–25 kg/m^2^, n (%	65 (74.7)
≥ 25 kg/m^2^, n (%	6 (6.9)
Use of hypnotics, n (%)	0 (0.0)

*BMI* Body mass index, *IQR* Interquartile range, *ODI* Oxygen desaturation index, *SD* Standard deviation

^a^Pre-pregnancy BMI

### Comparison of sleep problems during the second and third trimester of pregnancy

The percentage of women in the poor sleeper group increased from 34.1% in the second trimester to 62.5% in the third trimester (*p* <  0.001). More than 90% of women had DIS and/or DMS during the third trimester and the percentage of women with moderate to severe DIS and/or DMS increased from 31.8% in the second trimester to 55.7% in the third trimester (*p* <  0.001). Although the percentage of women having EDS (ESS ≥ 11) did not show significant changes from the second to the third trimester, almost half the participants did notice EDS during their pregnancy. The percentage of women with RLS/WED increased from 2.3 to 9.1% (*p* = 0.04). Although the percentage of women in the SDB group, which were those with a 3% ODI ≥ 5/h, showed an increasing tendency with values of 18.2% in the second trimester to 29.5% in the third trimester, the difference was not statistically significant (*p* = 0.08). There was no suspected moderate to severe SDB, defined as a 3% ODI ≥ 15/h, in the second trimester, and only one participant had a 3% ODI ≥ 15/h in the third trimester (Table 2).

**Table 2:** Comparison of body weight and sleep problems during the second and the third trimester of pregnancy

	Second trimester	Third trimester	*P* value^f^
Body mass index kg/m^2^, median (IQR)	22.6 (21.3–24.3)	24.2 (22.5–26.3)	< 0.001
Weight gain^a^ kg, median (IQR)	6.4 (4.8–8.1)	10.2 (8.5–13.3)	< 0.001
Rate of weight gain* %, median (IQR)	11.5 (7.7–13.9)	19.0 (14.9–22.6)	< 0.001
PSQI			
C1:sleep quality ≥2, n (%)	25 (28.4)	51 (58.0)	< 0.001
C2:sleep latency ≥2^b^, n (%)	26 (29.5)	43 (48.9)	0.003
C3:sleep duration ≥2^c^, n (%)	23 (26.1)	30 (34.1)	< 0.001
C4:sleep efficiency ≥2^d^, n (%)	5 (5.7)	12 (13.6)	0.10
C5:sleep disturbance ≥2, n (%)	23 (26.1)	49 (55.7)	< 0.001
C6:hypnotic use ≥2^e^, n (%)	0 (0.0)	1 (1.1)	< 0.001
C7:daytime dysfunction ≥2, n (%)	8 (9.1)	8 (9.1)	0.64
Global score ≥ 6, n (%)	30 (34.1)	55 (62.5)	< 0.001
ISI			
Mild to severe DIS, n (%)	36 (40.9)	71 (80.7)	< 0.001
Moderate to severe DIS, n (%)	16 (18.2)	37 (42.0)	< 0.001
Mild to severe DMS, n (%)	52 (59.1)	67 (76.1)	0.007
Moderate to severe DMS, n (%)	23 (26.1)	40 (45.5)	0.005
Mild to severe DIMS, n (%)	57 (64.8)	80 (90.9)	< 0.001
Moderate to severe DIMS, n (%)	28 (31.8)	49 (55.7)	< 0.001
Global score ≥ 10, n (%)	18 (20.5)	37 (42.0)	< 0.001
ESS ≥ 10, n (%)	46 (52.3)	47 (53.4)	1
ESS ≥ 11, n (%)	39 (44.3)	43 (48.9)	0.48
RLS/WED, n (%)	2 (2.3)	8 (9.1)	0.04
3% ODI ≥ 5/h, n (%)	16 (18.2)	26 (29.5)	0.08
5 ≤ 3% ODI < 15/h, n (%)	16 (18.2)	25 (28.4)	
3% ODI ≥ 15/h, n (%)	0 (0.0)	1 (1.1)	

*DIS* Difficulty initiating sleep, *DISM* Difficulty initiating and/or maintaining sleep, *DMS* Difficulty maintaining sleep, *ESS* Epworth Sleepiness Scale, *ISI* Insomnia Severity Index, *IQR* Interquartile range, *ODI* Oxygen desaturation index, *PSQI* Pittsburgh Sleep Quality Index global score, *RLS/WED* Restless legs syndrome/Willis–Ekbom disease

^a^From pre-pregnancy

^b^Sleep latency ≥31 min and the presence of difficulty initiating sleep

^c^Total sleep duration ≤6 h

^d^Sleep Efficiency < 85%

^e^More than one time

^f^Based on Wilcoxon signed-rank test for continuous variables or McNemar’s test for categorical data

### Sleep durations, insomnia, and excessive daytime sleepiness

The numbers of participants whose sleep duration in the third trimester was ≤6, 6–7, 7–8, 8–9, and > 9 h were 30, 27, 22, 6, and 3, respectively. In the group with sleep durations ≤6 h in the third trimester, decreased subjective sleep quality, moderate to severe DMS, higher insomnia severity, and EDS were observed (Additional file 2: Table S1). The median (IQR) total sleep duration in the third trimester was significantly shorter among participants with ISI values ≥10 than among those with ISI values < 10: 6.50 (6.00–7.75) hours vs. 7.00 (7.00–8.00) hours (*p* = 0.02). Furthermore, in the ESS ≥ 11 group in the third trimester, decreased subjective sleep quality, shorter sleep durations, and moderate to severe DMS were observed (Additional file 2: Table S2).

In a logistic regression analysis for EDS during the third trimester, the adjusted odds ratio (95% CI) of total sleep durations < 6 h, moderate to severe DMS, and 3% ODI ≥ 5/h were 3.25 (1.16–9.10), 4.74 (1.60–14.00), and 0.90 (0.28–2.89), respectively (Table 3). There was no collinearity between total sleep durations and DMS.

**Table 3:** Logistic regression analysis for daytime sleepiness during the third trimester of pregnancy

	Unadjusted odds ratio (95% confidence interval)	Adjusted odds ratio^a^ (95% confidence interval)
Total sleep duration		
> 6 h (reference)	1	1
≤ 6 h	3.82 (1.49–9.81)	3.25 (1.16–9.10)
Difficulty maintaining sleep		
None to mild (reference)	1	1
Moderate to severe	4.15 (1.70–10.10)	4.74 (1.60–14.00)
3% Oxygen desaturation index		
< 5/h (reference)	1	1
≥ 5/h	1.66 (0.66–4.18)	0.900.28–2.89)

^a^Adjusted for age and body mass index

### Sleep disordered breathing

The median BMI of the 3% ODI ≥ 5/h group in the third trimester was higher than that in the 3% ODI < 5/h group from pre-pregnancy to the third trimester (Table 4). DMS was frequent in the 3% ODI < 5/h group in the third trimester. However, associations with SDB and EDS was not found (Additional file 2: Table S3).

**Table 4:** Comparison of clinical characteristics between 3% ODI < 5/h group and 3% ODI ≥ 5/h group during the third trimester of pregnancy

	3% ODI < 5/h	3% ODI ≥ 5/h	*P* value ^*^
N	62	26	
Age years, mean ± SD	31.4 ± 4.6	29.7 ± 4.8	0.11
Primipara, n (%)	28 (45.9)	12 (46.2)	1
Body mass index			
Pre-pregnancy kg/m^2^, median (IQR)	20.0 (18.6–21.6)	21.5 (20.2–22.8)	0.03
Second trimester kg/m^2^, median (IQR)	22.2 (20.9–23.9)	23.8 (22.7–25.3)	0.01
Third trimester kg/m^2^, median (IQR)	23.6 (22.4–25.5)	26.3 (24.2–27.4)	0.004
Weight gain			
From pre-pregnancy kg, median (IQR)	10.0 (8.0–13.1)	10.8 (9.5–13.6)	0.22
From second trimester kg, median (IQR)	3.9 (2.5–4.4)	4.1 (3.1–5.0)	0.08
Rate of weight gain			
From pre-pregnancy %, median (IQR)	18.1 (13.8–23.4)	20.1 (18.5–21.7)	0.12
From second trimester %, median (IQR)	6.6 (4.5–8.5)	6.8 (5.8–7.9)	0.27

*IQR* Interquartile range, *ODI* Oxygen desaturation index, *SD* Standard deviation

*Based on unpaired *t*-test for normally distributed data, Mann-Whitney *U* test for non-normally distributed data, or Fisher’s exact test for categorical data

### Delivery outcomes and sleep/wake problems

Compared to the ISI < 10 group in the third trimester, the percentage of cesarean section was significantly higher in the ISI ≥ 10 group (*p* = 0.008), and the stillbirth case occurred in this group (Table 5). Short sleep durations, decreased subjective sleep quality, EDS, and SDB did not affect delivery outcomes or the infant’s condition (Additional file 2: Tables S4–S7).

**Table 5:** Insomnia during the third trimester of pregnancy and delivery outcomes

	ISI < 10	ISI ≥ 10	*P* value^b^
N	51	37^a^	
Gestational age months, median (IQR)	40.0 (39.4–40.7)	39.8 (38.9–40.6)	0.61
Duration of birth hours, median (IQR)	6.2 (4.3–9.7)	5.5 (3.5–9.1)	0.93
Type of birth			
Vaginal delivery, n (%)	50 (98.0)	29 (80.6)	0.008
Cesarean section, n (%)	1 (2.0)	7 (19.5)	
Labor induction, n (%)	6 (11.8)	5 (13.9)	0.76
Episiotomy, n (%)	0 (0.0)	1 (2.8)	0.41
Perineal laceration, n (%)	39 (76.5)	15 (41.7)	0.002
Volume of blood loss ml, median (IQR)	590 (370–850)	655 (485–1003)	0.25
Use of oxytocic, n (%)	29 (56.9)	24 (66.7)	0.38
Infant’s condition			
Alive, n (%)	51 (100.0)	35 (97.2)	0.41
Stillbirth, n (%)	0 (0.0)	1 (2.8)	
Weight of infant g, median (IQR)	3132 (2903–3399)	3235 (2955–3413)	0.59
Height of infant cm, median (IQR)	49.0 (48.0–50.2)	49.0 (48.0–50.0)	0.88
Apgar score			
1 min ≤ 6, n (%)	0 (0.0)	2 (5.7)	0.16
5 min ≤ 6, n (%)	0 (0.0)	1 (2.9)	0.41
Umbilical cord blood			
pH, median (IQR)	7.33 (7.28–7.37)	7.35 (7.32–7.39)	0.05
< 7.2, n (%)	3 (5.9)	1 (2.9)	0.64
≥ 7.2, n (%)	48 (94.1)	34 (97.1)	
PaCO_2_ mmHg, median (IQR)	34.9 (25.9–41.3)	31.4 (24.3–35.9)	0.17
< 32 mmHg, n (%)	20 (39.2)	20 (57.1)	0.19
32–68 mmHg, n (%)	30 (58.8)	15 (42.9)	
> 68 mmHg, n (%)	1 (2.0)	0 (0.0)	

*IQR* Interquartile range

^a^Uncertain one woman

^b^Based on Mann-Whitney *U* test for non-normally distributed data or Fisher’s exact test for categorical data

One case of stillbirth was registered in a 24-year-old primipara with gravidity 1. The course of pregnancy was normal until the onset of labor pain. She noticed a loss of fetal movement, and intrauterine fetal death was confirmed using cardiotocography. The woman had suffered from RLS/WED since she was 10 years old, and complained of DIS, DMS, and EDS during pregnancy. She had not been undergoing medical treatment for RLS/WED. Her sleep durations during pregnancy ranged from 7.5 to 8 h during the second trimester and 6 h during the third trimester. Her global PSQI, ISI, and ESS scores were 8, 12, and 15, respectively, in the second trimester and 12, 13, and 7, respectively, in the third trimester. The 3% ODI in the second and third trimesters were 1.9/h and 3.9/h, respectively. The weight of her infant was 3034 g, and coiling of the umbilical cord around the fetal neck was observed.

## Discussion

Even in pregnant women without gestational complications, sleep/wake problems were frequently observed and were more prevalent during the third trimester than during the second trimester, except for EDS. EDS was common throughout the course of pregnancy and was associated with short sleep durations and DMS, rather than SDB. Additionally, cesarean sections were slightly more prevalent in the insomnia group and one case of stillbirth with maternal RLS/WED occurred in this group. However, other sleep/wake problems did not affect delivery outcomes.

### Relationships among different sleep/wake problems in pregnant women

In this study, SDB was not a significant factor for EDS among pregnant women. It has been reported that ESS global scores at pre-pregnancy and in the third trimester are higher among pregnant women with obesity than among those without obesity [25]. Moreover, snoring during the first trimester is associated with not only continuous EDS throughout pregnancy, but also with EDS onset during pregnancy [5]. In the present study, only 6 participants were obese before pregnancy, and almost all participants suspected with SDB were considered mild cases. Such factors might however influence the relationship between SDB and EDS.

Although, DIS and DMS were frequently observed during the third trimester in the present study, moderate to severe DMS, rather than moderate to severe DIS, was associated with EDS. Physiological changes from the second to third trimester are thought to affect both DMS and EDS. During the third trimester, frequent urination due to a rapidly growing uterus, backpain, leg cramps, and increased fetal movement can cause both DIS and DMS. However, in a study reporting subjective findings using the Basic Nordic Sleep Questionnaire during the third trimester, while the percentage of participants with DIS was only 14%, the percentage of participants with DMS was as high as 70% [26]. As one of the causes of EDS among pregnant women without gestational complications, DMS due to physiological changes during late pregnancy may be important.

In the present study, short sleep durations of ≤6 h were associated with EDS. Total sleep durations were shorter among participants with insomnia than among those without insomnia. Although there was no collinearity between total sleep durations and DMS in a logistic regression analysis, shorter sleep durations induced by DMS, but might affect EDS.

Although an earlier meta-analysis associated shorter sleep durations with preterm birth [8], this was not confirmed in the present study. Shorter and longer sleep durations are known risk factors for obesity, diabetes, hypertension, and cardiovascular disease, which might be risk factors for gestational diabetes and hypertensive disorders during pregnancy [12, 27]. However, in pregnant women with normal gestational course until the second trimester, the effect of these factors on later gestational complication and delivery outcomes might be small. Along with sleep/wake problems and general sleep-related health problems, sleep durations have been speculated to affect gestational complications and delivery outcomes. We discuss potential problems in the one case of stillbirth, where the maternal sleep duration was 6 h in the third trimester, below (see section “Restless legs syndrome/Willis-Ekbom disease among pregnant women”).

### Sleep disordered breathing among pregnant women

In the present study, BMI values were higher in pregnant women with a 3% ODI ≥ 5/h than in those with a 3% ODI < 5/h from pre-pregnancy to the third trimester; the tendency was clearly noticeable in the later period of pregnancy. These findings are consistent with those of previous studies [10, 16, 28–30]. However, there were only few participants with obesity in this study. Additionally, a lower BMI is thought to be associated with a lower desaturation index obtained from the pulse oximeter.

SDB is associated with gestational complications, delivery outcomes, and neonatal conditions [6, 8–10, 13]. However, there was no significant effect on delivery outcomes and infant conditions in the 3% ODI ≥ 5/h group. Overall, SDB severity was relatively mild in the study participants; this is thought to have contribute to the good outcomes along with the fact that the participants had no gestational complications.

### Restless legs syndrome/Willis-Ekbom disease among pregnant women

RLS/WED is prevalent among pregnant women and affects DIS, DMS, and EDS [7, 11, 31]. In the present study, the prevalence of suspected RLS/WED was increased during the third trimester. However, the presence of RLS/WED did not affect EDS, insomnia symptoms, or subjective sleep quality. In pregnant Japanese women without gestational complications, few severe cases of RLS/WED have been reported, and almost all RLS/WED patients reported only mild to moderate symptoms [15]. Among 8 patients suspected with RLS/WED during the third trimester of pregnancy in this study, 7 reported moderate to severe symptoms. However, only 3 patients experienced these symptoms twice a week or more. The lower symptom occurrence frequency and the small sample size might have influenced the relationship between RLS/WED and EDS observed in this study.

It is worth mentioning that a stillbirth was observed in one pregnant woman with RLS/WED. This participant had complained of insomnia and EDS, which seemed to be related to her RLS/WED symptoms, and her subjective sleep quality had decreased. However, no other pregnancy-related complication was observed. While there are some reports of an association between RLS/WED and gestational complications [11, 32] as well as delivery outcomes [11, 14], other studies did not observe an association between RLS/WED and delivery outcomes [15, 31, 32]. Although we cannot assume an association between maternal sleep/wake problems and the stillbirth in this case observed here, further studies are needed to clarify the influence of RLS/WED on delivery outcomes.

The pathophysiological mechanism underlying the higher percentage of cesarean section in the insomnia group in this study is not clear. However, compared to pregnant women without RLS/WED, an odds ratio (95% CI) of 2.40 (1.03–4.42) for cesarean sections has been reported in pregnant Iranian women with RLS/WED, and insomnia has also been reported as being more prevalent among those with RLS/WED [14]. Although such a higher prevalence of cesarean sections in pregnant women with RLS/WED was not found in the present study, insomnia was related to cesarean section in previous reports as well as in the present study. To determine the relationship between cesarean sections and insomnia, a more comprehensive study on sleep/wake problems among pregnant women is needed.

### Limitations

Some limitations of the present study should be noted. First, we could not assess the specific sleep/wake problems of high-risk pregnant women who are also obese. Second, SDB was evaluated using only a pulse oximeter; therefore, no information on sleep stages, respiration itself, and position can be provided. Third, symptoms of RLS/WED were examined using a self-reported questionnaire, and the diagnosis of RLS/WED was not confirmed by sleep medicine specialists.

## Conclusions

From the second to the third trimester, an increase in sleep/wake problems was observed in pregnant Japanese women without complications. Moreover, EDS was associated with shorter sleep durations and DMS, rather than SDB. Moreover, there was one case of stillbirth with untreated RLS/WED, short sleep durations, insomnia, and EDS. However, our sample contained very few pregnant women with moderate to severe SDB, and there were no significant effects of SDB on delivery outcome and infant’s condition. Our findings indicate that comprehensive actions for sleep/wake problems among pregnant women, including education on sleep hygiene and/or medical treatment depending on specific requirement, are needed. To further evaluate the effects of sleep/wake disorders on gestational complications and delivery outcomes, additional large-scale research is needed, beginning from pre-pregnancy and including high-risk pregnant women.

## Supplementary information

**Additional file 1.** Data file.

**Additional file 2: Table S1.** Sleep durations in the third trimester of pregnancy and sleep problems. **Table S2.** Comparison of sleep problems between ESS < 11 group and ESS ≥ 11 group in the third trimester of pregnancy. **Table S3.** Comparison of sleep problems between 3% ODI < 5/h group and 3% ODI ≥ 5/h group in the third trimester of pregnancy. **Table S4.** Sleep durations in the third trimester of pregnancy and delivery outcomes. **Table S5.** Subjective sleep quality in the third trimester of pregnancy and delivery outcomes. **Table S6.** Excessive daytime sleepiness in the third trimester of pregnancy and delivery outcomes. **Table S7.** Sleep disordered breathing in the third trimester of pregnancy and delivery outcomes.

## Data Availability

All data generated or analyzed during this study are included in this published article and its supplementary information files.

## Abbreviations

AHI: Apnea-hypopnea index
BMI: Body mass index
CI: Confidence interval
CH-RLSq13: Cambridge-Hopkins questionnaire short form 13
DIS: Difficulty initiating sleep
DMS: Difficulty maintaining sleep
EDS: Excessive daytime sleepiness
ESS: Epworth Sleepiness Scale
IQR: Interquartile range
ISI: Insomnia Severity Index
ODI: Oxygen desaturation index
PSQI: Pittsburgh Sleep Quality Index
RLS/WED: Restless legs syndrome/Willis-Ekbom disease
SD: Standard deviation
SDB: Sleep disordered breathing
