# Supplementary material for: Characteristics of sleep/wake problems and delivery outcomes among pregnant Japanese women without gestational complications
Source: BMC Pregnancy Childbirth. 2020 Mar 20;20:179. doi: 10.1186/s12884-020-02868-1 (PMC7082997; doi:10.1186/s12884-020-02868-1)
Supplement: Supplementary file 2 — Additional file 2: Table S1. Sleep durations in the third trimester of pregnancy and sleep problems. Table S2. Comparison of sleep problems between ESS < 11 group and ESS ≥ 11 group in the third trimester of pregnancy. Table S3. Comparison of sleep problems between 3% ODI < 5/h group and 3% ODI ≥ 5/h group in the third trimester of pregnancy. Table S4. Sleep durations in the third trimester of pregnancy and delivery outcomes. Table S5. Subjective sleep quality in the third trimester of pregnancy and delivery outcomes. Table S6. Excessive daytime sleepiness in the third trimester of pregnancy and delivery outcomes. Table S7. Sleep disordered breathing in the third trimester of pregnancy and delivery outcomes. [file 12884_2020_2868_MOESM2_ESM.docx]

Table S1. Sleep durations in the third trimester of pregnancy and sleep problems

|  | | | Sleep durations < 6 | Sleep durations ≥ 6 | p value ^6)^ |
| --- | --- | --- | --- | --- | --- |
| N | | | 30 | 58 |  |
| Age years, mean ± SD | | | 31.1 ± 4.8 | 30.8 ± 4.6 | 0.80 |
| Primipara, n (%) | | | 15 (50.0) | 25 (43.9) | 0.65 |
| Body mass index kg/m^2^, median (IQR) | | | 20.0 (18.7–21.5) | 20.8 (19.0–22.6) | 0.11 |
| Weight gain ^1)^ kg, median (IQR) | | | 10.0 (8.9–12.4) | 10.5 (8.1–13.8) | 0.90 |
| Rate of weight gain* %, median (IQR) | | | 18.9 (17.2–22.7) | 19.2 (14.3–22.5) | 0.72 |
| PSQI | | |  |  |  |
|  | C1：sleep quality ≥ 2, n (%) | | 22 (73.3) | 29 (50.0) | 0.04 |
|  | C2：sleep latency ≥ 2 ^2)^, n (%) | | 17 (56.7) | 26 (44.8) | 0.37 |
|  | C3：sleep duration ≥ 2 ^3)^, n (%) | | 30 (100.0) | 0 (0.0) | < 0.001 |
|  | C4：sleep efficiency ≥ 2 ^4)^, n (%) | | 10 (33.3) | 2 (3.4) | < 0.001 |
|  | C5：sleep disturbance ≥ 2, n (%) | | 20 (66.7) | 29 (50.0) | 0.18 |
|  | C6：hypnotic use ≥ 2 ^5)^, n (%) | | 1 (3.3) | 0 (0.0) | 0.34 |
|  | C7：daytime dysfunction ≥ 2, n (%) | | 5 (16.7) | 3 (5.2) | 0.12 |
|  | Global score ≥ 6, n (%) | | 26 (86.7) | 29 (50.0) | < 0.001 |
| ISI | | |  |  |  |
|  | Mild to severe DIS, n (%) | | 25 (83.3) | 46 (79.3) | 0.78 |
|  | Moderate to severe DIS, n (%) | | 15 (50.0) | 22 (37.9) | 0.36 |
|  | Mild to severe DMS, n (%) | | 24 (80.0) | 43 (74.1) | 0.61 |
|  | Moderate to severe DMS, n (%) | | 19 (63.3) | 21 (36.2) | 0.02 |
|  |  | Mild to severe DIMS, n (%) | 27 (90.0) | 53 (91.4) | 1 |
|  |  | Moderate to severe DIMS, n (%) | 20 (66.7) | 29 (50.0) | 0.18 |
|  | Global score ≥ 10, n (%) | | 18 (60.0) | 19 (32.8) | 0.02 |
| ESS ≥ 11, n (%) | | | 21 (70.0) | 22 (37.9) | 0.007 |
| RLS/WED, n (%) | | | 3 (10.0) | 5 (8.6) | 1 |
| 3% ODI ≥ 5/hour, n (%) | | | 11 (36.7) | 15 (25.8) | 0.33 |
|  | 5 ≤ 3% ODI < 15/hour, n (%) | | 11 (36.7) | 14 (24.1) |  |
|  | 3% ODI ≥ 15/hour, n (%) | | 0 (0.0) | 1 (1.7) |  |

^1)^ From pre-pregnancy. ^2)^ Sleep latency ≥ 31 min and the presence of difficulty initiating sleep. ^3)^ Total sleep duration ≤I 6 hours. ^4)^ Sleep Efficiency < 85%. ^5)^ More than one time. ^6)^ Based on unpaired *t*-test for normally distributed data, Mann-Whitney *U* test for non-normally distributed data, or Fisher’s exact test for categorical data. DIS, difficulty initiating sleep; DISM, difficulty initiating and/or maintaining sleep; DMS, difficulty maintaining sleep; ESS, Epworth Sleepiness Scale; ISI, Insomnia Severity Index; IQR, interquartile range; ODI, oxygen desaturation index; PSQI, Pittsburgh Sleep Quality Index global score; RLS/WED, restless legs syndrome/Willis–Ekbom disease.

Table S2. Comparison of sleep problems between ESS < 11 group and ESS ≥ 11 group in the third trimester of pregnancy

|  | | | ESS < 11 | ESS ≥ 11 | p value ^5)^ |
| --- | --- | --- | --- | --- | --- |
| N | | | 45 | 43 |  |
| PSQI | | |  |  |  |
|  | C1：sleep quality ≥ 2, n (%) | | 21 (46.7) | 30 (69.8) | 0.03 |
|  | C2：sleep latency ≥ 2 ^1)^, n (%) | | 17 (37.8) | 26 (60.5) | 0.05 |
|  | C3：sleep duration ≥ 2 ^2)^, n (%) | | 9 (20.0) | 21 (48.8) | 0.007 |
|  | C4：sleep efficiency ≥ 2 ^3)^, n (%) | | 4 (8.9) | 8 (18.6) | 0.22 |
|  | C5：sleep disturbance ≥ 2, n (%) | | 18 (40.0) | 31 (72.1) | 0.003 |
|  | C6：hypnotic use ≥ 2 ^4)^, n (%) | | 0 (0.0) | 1 (2.3) | 0.49 |
|  | C7：daytime dysfunction ≥ 2, n (%) | | 1 (2.2) | 7 (16.3) | 0.03 |
|  | Global score, n (%) | | 21 (46.7) | 34 (79.1) | 0.002 |
| ISI | | |  |  |  |
|  | Mild to severe DIS, n (%) | | 32 (71.1) | 39 (90.7) | 0.03 |
|  | Moderate to severe DIS, n (%) | | 13 (28.9) | 24 (55.8) | 0.02 |
|  | Mild to severe DMS, n (%) | | 29 (64.4) | 38 (88.4) | 0.01 |
|  | Moderate to severe DMS, n (%) | | 13 (28.9) | 27 (62.8) | 0.003 |
|  |  | Mild to severe DIMS, n (%) | 39 (86.7) | 41 (95.3) | 0.27 |
|  |  | Moderate to severe DIMS, n (%) | 16 (35.6) | 33 (76.7) | <0.001 |
|  | Global score ≥ 10, n (%) | | 10 (22.2) | 27 (62.8) | <0.001 |
| RLS/WED, n (%) | | | 2 (4.4) | 6 (14.0) | 0.15 |
| 3% ODI ≥ 5/hour, n (%) | | | 11 (24.4) | 15 (34.9) | 0.35 |

^1)^ Sleep latency ≥ 31 min and the presence of difficulty initiating sleep. ^2)^ Total sleep duration ≤ 6 hours. ^3)^ Sleep Efficiency < 85%. ^4)^ More than one time. ^5)^ Based on Mann-Whitney U test for non-normally distributed data or Fisher’s exact test for categorical data. DIS, difficulty initiating sleep; DISM, difficulty initiating and/or maintaining sleep; DMS, difficulty maintaining sleep; ESS, Epworth Sleepiness Scale; ISI, Insomnia Severity Index; ODI, oxygen desaturation index; PSQI, Pittsburgh Sleep Quality Index global score; RLS/WED, restless legs syndrome/Willis–Ekbom disease.

Table S3. Comparison of sleep problems between 3% ODI < 5/hour group and 3% ODI ≥ 5/hour group in the third trimester of pregnancy

|  | | | 3% ODI < 5/hour | 3% ODI ≥ 5/hour | p value ^5)^ |
| --- | --- | --- | --- | --- | --- |
| N | | | 62 | 26 |  |
| PSQI | | |  |  |  |
|  | C1：sleep quality ≥ 2, n (%) | | 31 (50.0) | 20 (76.9) | 0.03 |
|  | C2：sleep latency ≥ 2 ^1)^, n (%) | | 28 (45.2) | 15 (57.7) | 0.35 |
|  | C3：sleep duration ≥ 2 ^2)^, n (%) | | 19 (30.6) | 11 (42.3) | 0.33 |
|  | C4：sleep efficiency ≥ 2 ^3)^, n (%) | | 6 (9.7) | 6 (23.1) | 0.17 |
|  | C5：sleep disturbance ≥ 2, n (%) | | 29 (46.8) | 20 (76.9) | 0.01 |
|  | C6：hypnotic use ≥ 2 ^4)^, n (%) | | 0 (0.0) | 1 (3.8) | 0.30 |
|  | C7：daytime dysfunction ≥ 2, n (%) | | 6 (9.7) | 2 (7.7) | 1 |
|  | Global score, n (%) | | 35 (56.5) | 20 (76.9) | 0.09 |
| ISI | | |  |  |  |
|  | Mild to severe DIS, n (%) | | 48 (77.4) | 23 (88.5) | 0.38 |
|  | Moderate to severe DIS, n (%) | | 23 (37.1) | 14 (53.8) | 0.16 |
|  | Mild to severe DMS, n (%) | | 46 (74.2) | 21 (80.8) | 0.59 |
|  | Moderate to severe DMS, n (%) | | 21 (33.9) | 19 (73.1) | 0.001 |
|  |  | Mild to severe DIMS, n (%) | 56 (90.3) | 24 (92.3) | 1 |
|  |  | Moderate to severe DIMS, n (%) | 29 (46.8) | 20 (76.9) | 0.01 |
|  | Global score ≥ 10, n (%) | | 22 (35.5) | 15 (57.7) | 0.06 |
| ESS ≥ 11, n (%) | | | 28 (45.2) | 15 (57.7) | 0.35 |
| RLS/WED, n (%) | | | 6 (9.7) | 2 (7.7) | 1 |

^1)^ Sleep latency ≥ 31 min and the presence of difficulty initiating sleep. ^2)^ Total sleep duration ≤ 6 hours. ^3)^ Sleep Efficiency < 85%. ^4)^ More than one time. ^5)^ Based on Mann-Whitney U test for non-normally distributed data or Fisher’s exact test for categorical data. DIS, difficulty initiating sleep; DISM, difficulty initiating and/or maintaining sleep; DMS, difficulty maintaining sleep; ESS, Epworth Sleepiness Scale; ISI, Insomnia Severity Index; ODI, oxygen desaturation index; PSQI, Pittsburgh Sleep Quality Index global score; RLS/WED, restless legs syndrome/Willis–Ekbom disease.

Table S4. Sleep durations in the third trimester of pregnancy and delivery outcomes

|  | | | Sleep durations ≤ 6 | Sleep durations > 6 | p value ^2)^ |
| --- | --- | --- | --- | --- | --- |
| N | | | 30 | 58 ^1)^ |  |
| Gestational age months, median (IQR) | | | 39.8 (39.0–40.6) | 40.0 (39.4–40.6) | 0.56 |
| Duration of birth hours, median (IQR) | | | 5.6 (4.6–9.2) | 6.3 (3.4–9.8) | 0.20 |
| Type of birth | | |  |  |  |
|  | Vaginal delivery, n (%) | | 26 (93.3) | 53 (88.1) | 0.44 |
|  | Cesarean section, n (%) | | 4 (6.7) | 4 (7.1) |  |
| Labor induction, n (%) | | | 5 (16.7) | 6 (10.5) | 0.50 |
| Episiotomy, n (%) | | | 0 (0.0) | 1 (1.8) | 1 |
| Perineal laceration, n (%) | | | 14 (46.7) | 40 (70.2) | 0.04 |
| Volume of blood loss ml, median (IQR) | | | 625 (340–898) | 600 (460–906) | 0.66 |
| Use of oxytocic, n (%) | | | 19 (63.3) | 34 (59.6) | 0.82 |
| Infant’s condition | | |  |  |  |
|  | Alive, n (%) | | 29 (96.7) | 57 (100.0) | 0.35 |
|  | Stillbirth, n (%) | | 1 (3.3) | 0 (0.0) |  |
| Weight of infant g, median (IQR) | | | 3042 (2953–3280) | 3234 (2906–3458) | 0.07 |
| Height of infant cm, median (IQR) | | | 49.0 (48.0–49.9) | 49.0 (48.0–50.0) | 0.88 |
| Apgar score | | |  |  |  |
|  | 1 min ≤ 6, n (%) | | 0 (0.0) | 2 (3.5) | 0.55 |
|  | 5 min ≤ 6, n (%) | | 0 (0.0) | 1 (1.8) | 1 |
| Umbilical cord blood | | |  |  |  |
|  | pH, median (IQR) | | 7.34 (7.30–7.38) | 7.34 (7.29–7.39) | 0.93 |
|  |  | < 7.2, n (%) | 2 (6.9) | 2 (3.5) | 0.60 |
|  |  | ≥ 7.2, n (%) | 27 (93.1) | 55 (96.5) |  |
|  | PaCO_2_ mmHg, median (IQR) | | 32.3 (27.8–40.7) | 32.8 (24.5–38.6) | 0.46 |
|  |  | < 32 mmHg, n (%) | 14 (48.3) | 26 (45.6) | 0.39 |
|  |  | 32-68 mmHg, n (%) | 14 (48.3) | 31 (54.4) |  |
|  |  | > 68 mmHg, n (%) | 1 (3.4) | 0 (0.0) |  |

^1)^ Uncertain one woman. ^2)^ Based on Mann-Whitney U test for non-normally distributed data or Fisher’s exact test for categorical data. IQR, interquartile range.

Table S5. Subjective sleep quality in the third trimester of pregnancy and delivery outcomes

|  | | | PSQI GS < 6 | PSQI GS ≥ 6 | p value ^2)^ |
| --- | --- | --- | --- | --- | --- |
| N | | | 33 | 54 ^1)^ |  |
| Gestational age months, median (IQR) | | | 40.0 (38.9–40.6) | 40.0 (39.4–40.7) | 0.56 |
| Duration of birth hours, median (IQR) | | | 6.1 (3.4–9.7) | 6.0 (4.3–9.1) | 0.54 |
| Type of birth | | |  |  |  |
|  | Vaginal delivery, n (%) | | 30 (90.9) | 49 (90.7) | 1 |
|  | Cesarean section, n (%) | | 3 (9.1) | 5 (9.3) |  |
| Labor induction, n (%) | | | 5 (15.2) | 6 (11.1) | 0.74 |
| Episiotomy, n (%) | | | 0 (0.0) | 1 (1.9) | 1 |
| Perineal laceration, n (%) | | | 26 (78.8) | 25 (51.9) | 0.01 |
| Volume of blood loss ml, median (IQR) | | | 580 (370–950) | 630 (460–888) | 0.90 |
| Use of oxytocic, n (%) | | | 18 (54.5) | 35 (64.8) | 0.37 |
| Infant’s condition | | |  |  |  |
|  | Alive, n (%) | | 33 (100) | 53 (98.1) | 1 |
|  | Stillbirth, n (%) | | 0 (0.0) | 1 (1.9) |  |
| Weight of infant g, median (IQR) | | | 3126 (2776–3374) | 3207 (3005–3445) | 0.10 |
| Height of infant cm, median (IQR) | | | 49.0 (48.0–50.0) | 49.0 (48.0–50.0) | 0.32 |
| Apgar score | | |  |  |  |
|  | 1 min ≤ 6, n (%) | | 1(3.0) | 1 (1.9) | 1 |
|  | 5 min ≤ 6, n (%) | | 1(3.0) | 0 (0.0) | 0.38 |
| Umbilical cord blood | | |  |  |  |
|  | pH, median (IQR) | | 7.32 (7.28–7.37) | 7.34 (7.31–7.39) | 0.19 |
|  |  | < 7.2, n (%) | 2 (6.1) | 2 (3.8) | 0.64 |
|  |  | ≥ 7.2, n (%) | 31 (93.9) | 51 (96.2) |  |
|  | PaCO_2_ mmHg, median (IQR) | | 34.9 (27.8–41.1) | 31.4 (23.2–37.5) | 0.14 |
|  |  | < 32 mmHg, n (%) | 12 (36.4) | 28 (52.8) | 0.18 |
|  |  | 32-68 mmHg, n (%) | 21 (63.6) | 24 (45.3) |  |
|  |  | > 68 mmHg, n (%) | 0 (0.0) | 1 (1.9) |  |

^1)^ Uncertain one woman. ^2)^ Based on Mann-Whitney U test for non-normally distributed data or Fisher’s exact test for categorical data. IQR, interquartile range.

Table S6. Excessive daytime sleepiness in the third trimester of pregnancy and delivery outcomes

|  | | | ESS < 11 | ESS ≥ 11 | p value ^3)^ |
| --- | --- | --- | --- | --- | --- |
| N | | | 45 | 43 ^1)^ |  |
| Gestational age months, median (IQR) | | | 40.0 (39.1–40.6) | 40.0 (39.2–40.7) | 0.46 |
| Duration of birth hours, median (IQR) | | | 5.7 (3.4–8.9) | 6.0 (4.7–10.9) | 0.20 |
| Type of birth | | |  |  |  |
|  | Vaginal delivery, n (%) | | 42 (93.3) | 37 (88.1) | 0.63 |
|  | Cesarean section, n (%) | | 3 (6.7) | 5 (7.1) |  |
| Labor induction, n (%) | | | 6 (13.3) | 5 (11.9) | 1 |
| Episiotomy, n (%) | | | 1 (2.2) | 0 (0.0) | 1 |
| Perineal laceration, n (%) | | | 31 (68.9) | 23 (54.8) | 0.19 |
| Volume of blood loss ml, median (IQR) | | | 620 (370–910) | 595 (470–827) | 0.74 |
| Use of oxytocic, n (%) | | | 25 (55.6) | 28 (66.7) | 0.38 |
| Infant’s condition | | |  |  |  |
|  | Alive, n (%) | | 44 (97.8) | 42 (100.0) | 1 |
|  | Stillbirth, n (%) | | 1 (2.2) ^2)^ | 0 (0.0) |  |
| Weight of infant g, median (IQR) | | | 3132 (2894–3404) | 3165 (2958–3399) | 0.53 |
| Height of infant cm, median (IQR) | | | 49.0 (48.0–50.0) | 49.0 (48.0–50.0) | 0.17 |
| Apgar score | | |  |  |  |
|  | 1 min ≤ 6, n (%) | | 1(2.3) | 1 (2.4) | 1 |
|  | 5 min ≤ 6, n (%) | | 0(0.0) | 1 (2.4) | 0.48 |
| Umbilical cord blood | | |  |  |  |
|  | pH, median (IQR) | | 7.33 (7.30–7.37) | 7.34 (7.30–7.39) | 0.37 |
|  |  | < 7.2, n (%) | 1 (2.3) | 3 (7.1) | 0.36 |
|  |  | ≥ 7.2, n (%) | 43 (97.7) | 39 (92.9) |  |
|  | PaCO_2_ mmHg, median (IQR) | | 32.5 (26.1–39.8) | 32.4 (24.4–37.5) | 0.94 |
|  |  | < 32 mmHg, n (%) | 19 (43.2) | 21 (50.0) | 0.36 |
|  |  | 32-68 mmHg, n (%) | 25 (56.8) | 20 (47.6) |  |
|  |  | > 68 mmHg, n (%) | 0 (0.0) | 1 (2.4) |  |

^1)^ Uncertain one woman. ^2)^ At the second trimester, this participant’s ESS score was 15. ^3)^ Based on Mann-Whitney U test for non-normally distributed data or Fisher’s exact test for categorical data. IQR, interquartile range.

Table S7. Sleep disordered breathing in the third trimester of pregnancy and delivery outcomes

|  | | | 3% ODI < 5/hour | 3% ODI ≥ 5/hour | p value ^2)^ |
| --- | --- | --- | --- | --- | --- |
| N | | | 61 ^1)^ | 26 |  |
| Gestational age months, median (IQR) | | | 40.0 (39.6–40.3) | 39.7 (39.2–40.2) | 0.48 |
| Duration of birth hours, median (IQR) | | | 5.9 (4.0–10.3) | 6.1 (4.0–9.1) | 0.93 |
| Type of birth | | |  |  |  |
|  | Vaginal delivery, n (%) | | 57 (93.4) | 22 (84.6) | 0.35 |
|  | Cesarean section, n (%) | | 4 (6.6) | 4 (15.4) |  |
| Labor induction, n (%) | | | 8 (13.1) | 3 (11.5) | 0.84 |
| Episiotomy, n (%) | | | 0 | 1 (3.8) | 0.13 |
| Perineal laceration, n (%) | | | 40 (65.6) | 14 (53.8) | 0.31 |
| Volume of blood loss ml, median (IQR) | | | 580 (370–800) | 735 (460–910) | 0.27 |
| Use of oxytocic, n (%) | | | 37 (60.7) | 16 (61.5) | 0.94 |
| Infant’s condition | | |  |  |  |
|  | Alive, n (%) | | 60 (96.8) | 26 (100.0) | 1 |
|  | Stillbirth, n (%) | | 1 (1.6) | 0 |  |
| Weight of infant g, median (IQR) | | | 3176 (2922–3404) | 3145 (2914–3445) | 0.51 |
| Height of infant cm, median (IQR) | | | 49.0 (48.0–50.2) | 49.0 (47.5–49.5) | 0.51 |
| Apgar score | | |  |  |  |
|  | 1 min ≤ 6, n (%) | | 1(1.6) | 2 (7.7) | 0.15 |
|  | 5 min ≤ 6, n (%) | | 1(1.6) | 1 (3.8) | 0.52 |
| Umbilical cord blood | | |  |  |  |
|  | pH, median (IQR) | | 7.32 (7.28–7.37) | 7.36 (7.32–7.41) | 0.06 |
|  |  | < 7.2, n (%) | 3 (5.0) | 1 (3.8) | 1 |
|  |  | ≥ 7.2, n (%) | 57 (95.0) | 25 (96.2) |  |
|  | PaCO_2_ mmHg, median (IQR) | | 34.8 (26.8–41.3) | 29.5 (22.8–32.8) | 0.15 |
|  |  | < 32 mmHg, n (%) | 24 (40.0) | 16 (61.5) | 0.14 |
|  |  | 32-68 mmHg, n (%) | 35 (58.3) | 10 (38.5) |  |
|  |  | > 68 mmHg, n (%) | 1 (1.7) | 0 |  |

^1)^ Uncertain one woman. ^2)^ Based on Mann-Whitney U test for non-normally distributed data or Fisher’s exact test for categorical data. IQR, interquartile range.
